# Telemedicine and Access to Elective Cholecystectomy for Socially Vulnerable Adults: A Pilot Randomized Clinical Trial

**DOI:** 10.1001/jamanetworkopen.2024.38137

**Published:** 2024-10-09

**Authors:** Emna Bakillah, Sean Harbison, Francis E. Rosato, Maria S. Altieri, Jon B. Morris, Elinore Kaufman, Marilyn Schapira, MaryAnne Peifer, Rachel R. Kelz

**Affiliations:** 1 Department of Surgery, Perelman School of Medicine of the University of Pennsylvania, Philadelphia; 2 Department of Surgery, Center for Surgery and Health Economics, Philadelphia, Pennsylvania; 3 Leonard Davis Institute of Health Economics, University of Pennsylvania, Philadelphia; 4 Department of Internal Medicine, Perelman School of Medicine of the University of Pennsylvania, Philadelphia

## Abstract

**Importance:**

Socially vulnerable patients with symptomatic cholelithiasis are more likely to face barriers to accessing surgical care. This barrier to access can lead to delays in treatment, the need for emergent cholecystectomy, and worse outcomes.

**Objectives:**

To determine the effectiveness of telemedicine vs in-person surgical consultation on access to elective cholecystectomy in socially vulnerable populations and to evaluate the association of scheduling navigation with access to elective cholecystectomy in these populations.

**Design, Setting, and Participants:**

This pilot randomized clinical trial conducted in a single academic center enrolled 60 adults from February 1, 2023, to February 21, 2024, with 3-month follow-up of clinical outcomes. Data were also collected retrospectively on a comparison group of 32 patients referred from June 30 to December 29, 2022. Adults with social vulnerability, such as being non-White or Hispanic or having nonprivate insurance or low income, with a diagnosis of symptomatic cholelithiasis and referral for outpatient surgical consultation were included.

**Interventions:**

All trial participants were randomized to the telemedicine or in-person surgical consultation group, and received professional scheduling navigation. The latter intervention was compared with a historical cohort without navigation assistance.

**Main Outcomes and Measures:**

The primary outcome was completion of outpatient surgical consultation. Secondary outcomes included receipt of treatment and operative urgency.

**Results:**

The trial enrolled 60 participants (30 per arm). Their mean (SD) age was 48.2 (18.2) years, 50 (83.3%) were female, 2 (3.3%) were Asian, 39 (65.0%) were Black, 8 (13.3%) were Hispanic, 11 (18.3%) were White, and 41 (68.3%) had no private insurance. The historical patient cohort included 32 participants (mean [SD] age, 45.9 [3.2] years; 27 [84.4%] female; 3 [9.4%] Asian, 15 [46.9%] Black, 10 [31.3%] Hispanic, and 6 [18.8%] White; and 18 [56.3%] without private insurance). In total, 18 trial participants assigned to telemedicine (60.0%) completed surgical consultations compared with 23 trial participants assigned to in-person visits (76.7%; *P* = .17). For telemedicine participants who underwent cholecystectomy, 3 of 7 (42.9%) underwent emergent cholecystectomy compared with 0 of 14 (0%) participants with in-person consultations (*P* = .03). Of 30 trial participants who received scheduling navigation, 23 (76.7%) completed surgical consultations compared with 15 of 32 patients in the historical cohort who did not receive scheduling navigation (46.9%; *P* = .02). Of 14 trial participants who received scheduling navigation and cholecystectomy, no participants underwent emergent cholecystectomy compared with 4 of 16 (25.0%) participants in the historical cohort without scheduling navigation (*P* = .04).

**Conclusions and Relevance:**

In this pilot randomized clinical trial of socially vulnerable adults with symptomatic cholelithiasis, telemedicine consultation compared with in-person visits did not improve access to elective outpatient surgical care. However, scheduling navigation services may improve access to elective outpatient surgical care. Future large-scale studies are needed to identify possible barriers to virtual health care and mechanisms to address inequities.

**Trial Registration:**

ClincialTrials.gov Identifier: NCT05745077

## Key Points

**Question:**

Are telemedicine surgical consultation visits or scheduling navigation services associated with improved access to elective cholecystectomy in socially vulnerable populations?

**Findings:**

In this pilot randomized clinical trial with 60 participants, patients assigned to telemedicine were not significantly more likely than patients with in-person visits to complete surgical consultation. However, trial patients with scheduling navigation services were more likely than 32 patients from a retrospective observational cohort without navigation assistance to complete surgical consultation (76.7% vs 46.9%).

**Meaning:**

For socially vulnerable communities, telemedicine consultations may not provide adequate access to elective surgical care; however, scheduling navigation services may be associated with outpatient surgical care.

## Teaser

This pilot randomized clinical trial compares the effectiveness of telemedicine consultation with in-person visits and the association of scheduling navigation assistance vs no assistance with improving access to elective outpatient surgical care among socially vulnerable adults with symptomatic cholelithiasis.

## Introduction

Symptomatic cholelithiasis is a common diagnosis effectively treated with laparoscopic cholecystectomy. Laparoscopic cholecystectomy can be safely performed electively in the outpatient setting.^1^ Delayed treatment for symptomatic cholelithiasis often results in acute cholecystitis and related diseases that necessitate emergency surgery.^2^ Emergent cholecystectomy results in worse outcomes, with higher rates of conversion from a laparoscopic to an open procedure, increased hospital length of stay, and increased rates of morbidity and mortality, when compared with elective cholecystectomy.^3,4^

Socially vulnerable patients, such as Black and Hispanic individuals, non-English primary language speakers, and individuals living in areas of greater social vulnerability, are at increased risk of treatment delays and emergent cholecystectomy.^5,6,7,8^ For historically marginalized communities, patient- and system-level barriers to timely outpatient surgical consultation represent 1 source of delay.^9^ For example, work conflicts, limited paid time off, long clinic wait times, and limited ability to contact the surgical clinic often preclude the ability of patients with social vulnerabilities to seek a consultation or receive elective operative care.^10,11,12,13^

Telemedicine and professional navigation services have previously been shown to address barriers to access to care.^14,15^ The aims of the present study were (1) to determine the effectiveness of telemedicine surgical consultation (compared with in-person consultation) on access to outpatient elective cholecystectomy in socially vulnerable populations and (2) to assess the association of scheduling navigation with access to outpatient elective cholecystectomy in these populations. We hypothesized that both interventions would improve access to outpatient elective surgery.

## Methods

### Study Overview

We conducted a pilot randomized clinical trial of the intervention of preoperative telemedicine consultation for socially vulnerable patients referred to a surgeon for their diagnosis of symptomatic cholelithiasis within a single academic institution (University of Pennsylvania Health System) between February 1, 2023, and February 21, 2024. The study protocol was reviewed and approved by the Institutional Review Board at the University of Pennsylvania, is registered at ClinicalTrials.gov, and is provided in Supplement 1. The study followed the Consolidated Standards of Reporting Trials (CONSORT) reporting guideline extension for randomized pilot and feasibility trials.^16^ Verbal and digital written informed consent were provided at the time of enrollment.

### Trial Participants, Recruitment Strategy, and Randomization

Eligible patients were screened using the electronic health record (EHR) by searching for diagnostic imaging reports and consult orders. Adult patients were eligible if they (1) had a diagnosis of symptomatic cholelithiasis, (2) had a documented referral for outpatient surgical consultation, (3) were socially vulnerable as defined by 1 or more of the criteria adapted from the Centers for Disease Control and Prevention,^17^ namely, being non-White or Hispanic or having nonprivate insurance or low income, (4) could be reached by telephone and spoke English, and (5) provided informed consent. Race and ethnicity were self-reported and extracted from the EHR. They were assessed in this study as part of the definition of social vulnerability, and were categorized for this study as being Asian, Black, Hispanic, non-Hispanic, White, other or unknown. Patients were excluded if they already had a scheduled surgical consultation or cholecystectomy. Patients who provided consent were randomized in parallel by week in a 1:1 ratio to either telemedicine or in-person surgical consultation. Participants were financially compensated (US $20) at the time of surgical consultation.

### Interventions

#### Telemedicine Consultation

Telemedicine appointments were scheduled through the electronic health portal (MyPennMedicine) and participants received digital written instructions through their email or portal. Telemedicine appointments were conducted using the BlueJeans video software (Verizon) or Epic Video Client (Epic Systems Corporation), which are uniformly used for telehealth throughout the University of Pennsylvania Health System. Each telemedicine appointment was scheduled for a standard 30-minute new consultation visit, which is the same length of time assigned to in-person consultation visits. Study participants assigned to telemedicine consultations were compared with the current standard, which is in-person surgical consultation.

#### Scheduling Navigation

All study participants were recruited by 1 member of the research team (E.B.) by phone call. After obtaining consent, 1 administrative team member from the outpatient surgical clinic was added to the same phone call to schedule the patient for their appointment. If the administrative team member was unavailable, a secure message was sent to ask the member to call the patient at a future time to schedule the appointment.

To evaluate scheduling navigation, a retrospective cohort of patients meeting study inclusion criteria who did not have scheduling navigation assistance were identified in the EHR (June 3 to December 29, 2022). These patients did not receive any contact from the health care system regarding scheduling outpatient surgical consultation after their surgical referral was ordered. Study participants assigned to in-person consultation were compared with the historical cohort.

### Outcome Measures

Data were collected via the EHR up to 3 months from the time of recruitment. Outcome measures were defined as the milestones along the clinical pathway from diagnosis to treatment. The primary outcome was completion of outpatient surgical consultation. Secondary outcomes included treatment recommendation, scheduling of treatment, and receipt of treatment. Time from referral to milestone acquisition, operative urgency, and related emergency department visits after surgical referral were recorded.

### Statistical Analysis

#### Sample Size

Sample size was calculated based on the χ^2^ test of the primary outcome, which was the difference in the percentages of patients who completed outpatient surgical consultations between the telemedicine and in-person groups. Assuming a 2-tailed α of .05 and a standardized effect size of 0.40, 25 participants were required per group to achieve a power of 0.80.

#### Data Analysis

Descriptive statistics were summarized using mean (SD) values for continuous variables and counts and percentages for categorical variables. Univariate analyses were performed using χ^2^ analysis and 2-sample *t* tests. All *P* values were from 2-sided tests, and results were deemed statistically significant at *P* < .05. All analyses were performed in Stata, version 17.0 (StataCorp LLC).

## Results

### Baseline Characteristics

We assigned 30 participants to telemedicine and 30 to in-person consultations (Figure). In the trial cohort, the mean (SD) age was 48.2 (18.2) years, 50 (83.3%) were female, 10 (16.7) were male, 2 (3.3%) were Asian, 39 (65.0%) were Black, 8 (13.3%) were Hispanic, and 11 (18.3%) were White (Table 1). With regard to insurance status, 19 participants (31.7%) had private insurance, and 41 participants (68.3%) had no private insurance. The historical cohort of patients included 32 participants (mean [SD] age, 45.9 [3.2] years; 27 [84.4%] female and 5 [15.6%] male; 3 [9.4%] Asian, 15 [46.9%] Black, 10 [31.3%] Hispanic, and 6 [18.8%] White; and 18 [56.3%] without private insurance).

**Figure.**
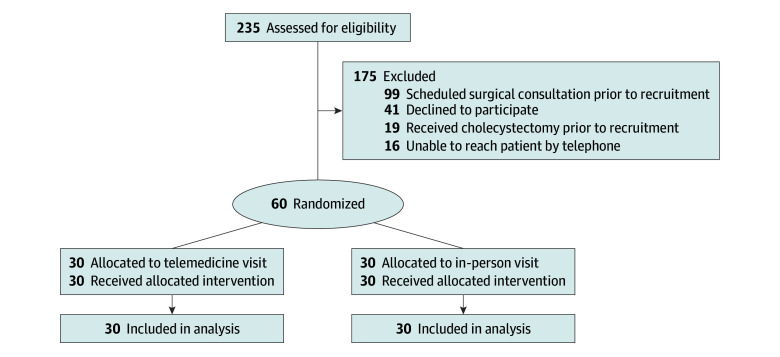
CONSORT Flow Diagram

**Table 1.** Baseline Characteristics

Covariate	Patients, No. (%)		
	Enrolled in randomized clinical trial		In historical cohort
	Telemedicine with navigation (n = 30)	In person with navigation (n = 30)	In person without navigation (n = 32)
Age, mean (SD), y	46.2 (3.5)	50.2 (3.1)	45.9 (3.2)
Sex			
Female	26 (86.7)	24 (80.0)	27 (84.4)
Male	4 (13.3)	6 (20.0)	5 (15.6)
Race			
Asian	0 (0)	2 (6.7)	3 (9.4)
Black	20 (66.7)	19 (63.3)	15 (46.9)
White	8 (26.7)	3 (10.0)	6 (18.8)
Other^a^	1 (3.3)	2 (6.7)	6 (18.8)
Unknown	1 (3.3)	4 (13.3)	2 (6.3)
Ethnicity			
Hispanic	3 (10.0)	5 (16.7)	10 (31.3)
Non-Hispanic	27 (90.0)	25 (83.3)	22 (68.7)
Insurance			
Private	9 (30.0)	10 (33.3)	14 (43.8)
Medicare	9 (30.0)	9 (30)	4 (12.5)
Medicaid	12 (40.0)	11 (36.7)	12 (37.5)
None	0 (0)	0 (0)	2 (6.3)
Diagnostic environment			
Primary care	10 (33.3)	17 (56.7)	21 (65.6)
Emergency department	20 (66.7)	13 (43.3)	11 (34.4)
Diagnostic imaging			
Ultrasonography	22 (73.3)	26 (86.7)	26 (81.3)
Computed tomography	6 (20.0)	3 (10.0)	5 (15.6)
Magnetic resonance	2 (6.7)	1 (3.3)	1 (3.1)

^a^ Race and ethnicity listed as “other” in the electronic health record.

### Outcomes

#### Telemedicine vs In-Person Visits

In total, 18 participants assigned to telemedicine visits (60.0%) completed surgical consultations compared with 23 participants assigned to in-person visits (76.7%; *P* = .17). Of the participants who received cholecystectomy, 3 of 7 participants (42.9%) assigned to telemedicine visits underwent emergent cholecystectomy compared with none of the 14 participants assigned to in-person visits (*P* = .03) (Table 2). There were no statistically significant differences in any of the other secondary outcomes by visit type. There was no difference in the mean number of days from referral to scheduled surgical consultation between the telemedicine and in-person groups, (34.3 vs 34.6, *P* = .98). Documented reasons for failure to complete each milestone are recorded in Table 3. Of 30 patients in the telemedicine group, 6 did not show up to their surgical consultation compared with 1 of 30 patients in the in-person group.

**Table 2.** Quantitative Outcomes

Care progression milestone	Patients, No./total No. (%)				
	Enrolled in randomized clinical trial			In historical cohort	
	Telemedicine with navigation (n = 30)	In person with navigation (n = 30)	*P* value^a^	In person without navigation (n = 32)	*P* value^b^
Completed surgical consultation	18/30 (60.0)	23/30 (76.7)	.17	15/32 (46.9)	.02
Time from referral to scheduled surgical consultation, mean (SD), d	34.3 (7.8)	34.6 (9.4)	.98	42.1 (9.9)	.72
Patients who completed surgical consultation					
Cholecystectomy recommended	13/18 (72.2)	20/23 (86.9)	.27	12/15 (80.0)	.57
Patients who were recommended cholecystectomy					
Cholecystectomy scheduled	8/13 (61.6)	17/20 (85.0)	.21	12/12 (100)	.16
Patients scheduled for cholecystectomy					
Elective cholecystectomy performed	4/8 (50.0)	14/17 (82.4)	.16	12/12 (100)	.12
Time from surgical consultation to performed elective cholecystectomy, mean (SD), d	27.8 (15.8)	40.8 (9.8)	.51	25.7 (11.9)	.38
ED visit after referral	6/30 (20.0)	6/30 (20.0)	>.99	6/32 (18.7)	.90
Patients who underwent cholecystectomy					
Emergent cholecystectomy	3/7 (42.9)	0/14 (0)	.03	4/16 (25.0)	.04

Abbreviation: ED, emergency department.

^a^ Comparing telemedicine with in-person visits.

^b^ Comparing with vs without navigation assistance.

**Table 3.** Documented Reasons for Failure to Progress in Milestone Acquisition

Care progression milestone and documented reason for progression failure	Patients, No.		
	Enrolled in randomized clinical trial		In historical cohort
	Telemedicine with navigation (n = 30)	In person with navigation (n = 30)	In person without navigation (n = 32)
Completed surgical consultation			
No show	6	1	NA
Cancellation	4	6	NA
Emergent cholecystectomy prior to visit	2	0	NA
Not scheduled	NA	NA	17
Cholecystectomy recommended			
Symptoms inconsistent with biliary colic	4	2	2
High risk	1	0	0
No documented reason	0	1	0
Required further workup	0	0	1
Cholecystectomy scheduled			
Required further workup	2	2	0
Patient preferred watchful waiting	2	1	0
No documented reason	1	0	0
Elective cholecystectomy performed			
Cancellation	2	2	0
Positive COVID test	1	0	0
Emergent cholecystectomy prior to scheduled operation	1	0	0
No show	0	1	0
Positive pregnancy test	0	1	0

Abbreviation: NA, not applicable.

#### Navigation Assistance vs No Assistance

Of 30 trial participants with navigation assistance, 23 (76.7%) completed surgical consultations compared with 15 of 32 patients in the historical cohort (46.9%) who completed surgical consultations without navigation assistance (*P* = .02). Furthermore, among patients who received cholecystectomy, 0 of 14 trial participants scheduled using navigation assistance required emergent cholecystectomy compared with 4 of 16 patients in the historical cohort (25.0%) scheduled without navigation assistance (*P* = .04) (Table 2).

## Discussion

In this pilot randomized clinical trial of socially vulnerable adults with symptomatic cholelithiasis, telemedicine consultation did not improve access to elective outpatient surgical care compared with in-person visits. The comparable time from referral to scheduled surgical consultation between these 2 groups suggests that delays are not the driver of disparities in access to elective surgical care. Further, the use of telemedicine was not effective in mitigating this disparity in our cohort of socially vulnerable patients. This may be due to limited digital literacy and lack of reliable internet service or devices.^18,19^ This hypothesis is supported by the high no-show rate in our studied telemedicine group. This finding highlights the need to test new ways to connect socially vulnerable people to surgical care, digitally, in person, or otherwise. Future work should include direct engagement with vulnerable individuals to determine best approaches to streamline their care.

In contrast to our result that telemedicine consultations did not improve access to elective outpatient surgical care, our findings suggest that the use of professional scheduling navigation has the potential to improve access to outpatient surgical consultation and elective cholecystectomy compared with no navigation assistance. This finding is consistent with the previous literature that reports improved compliance rates in attending specialist appointments when professional navigation is readily accessible to patients.^20,21^ Our study supports quality improvement efforts targeted at the level of the health care system to expand the use of scheduling navigation for outpatient surgical consultation.

### Limitations

This study has several limitations. First, our study did not include patients who lacked access to the health care system; thus, our results may underestimate the association of scheduling navigation with improved access to elective cholecystectomy. Second, individuals whose primary language is not English were excluded from recruitment; however, some socially vulnerable patients have limited English proficiency, which may limit the generalizability of our findings. Third, the comparator group without navigation assistance was a retrospective cohort, which may result in residual confounding on the outcomes associated with scheduling navigation. Last, this was a pilot study with a small number of enrolled participants, which could limit the power to detect significant differences.

## Conclusions

In conclusion, findings from this pilot randomized clinical trial suggest that telemedicine consultation visits may not be effective for connecting socially vulnerable patients to outpatient surgical care and elective surgery. On the other hand, standardizing methods of scheduling navigation may be associated with improved access to outpatient elective surgical care. Future large-scale studies are needed to identify possible barriers to virtual health care and mechanisms to address inequities.

## Notes

Supplement 1.
Trial Protocol

Supplement 2.
Data Sharing Statement
