# Supplementary material for: Telemedicine and Access to Elective Cholecystectomy for Socially Vulnerable Adults: A Pilot Randomized Clinical Trial
Source: JAMA Netw Open. 2024 Oct 9;7(10):e2438137. doi: 10.1001/jamanetworkopen.2024.38137 (PMC11581524; doi:10.1001/jamanetworkopen.2024.38137)
Supplement: Supplement 1. — Trial Protocol [file jamanetwopen-e2438137-s001.pdf]

Using Data to Achieve Surgical Health Equity in the Community

Principal Investigator: Rachel Kelz, MD

IRB# 852524

Study Protocol

Version Date: 11/22/2022

PROJECT ABSTRACT

Gallbladder disease (GBD) is extremely common, affecting 10% to 20% of adults in the United States with a prevalence as high as 70% in specific ethnic groups. Delayed treatment often leads to emergency surgery. Emergent cholecystectomy results in increased morbidity, mortality, length of stay, and higher costs when compared to elective cholecystectomy. Black and Hispanic patients, non-English primary language speakers, and those living in areas of greater social vulnerability are at increased risk of emergent GB surgery. Black patients with established primary care providers within the Penn Medicine Primary Care Service Line are more likely to undergo emergent (vs elective) cholecystectomy than White patients (Black: 65% (76/117); White, 41% (125/303); $p<0.001$). To address this disparity, we propose the following specific aims: 1) To examine racial disparities of patients with symptomatic GBD with attention to conditional effects of social vulnerabilities (SV), and 2) to compare the effectiveness of telemedicine consultation for symptomatic GBD in patients with SV to usual care. The first aim will be an observational study of Penn Medicine data using regression to adjust for potential confounders, and interaction terms to examine the conditional effects of SV factors. For aim 2, we will perform a pilot study of telemedicine consultation. These results will provide data to develop evidence-based solutions to disparities within Penn Medicine and to serve as preliminary data for a subsequent R01 or PCORI grant to address surgical disparities in patients with symptomatic GBD.

PROJECT DESCRIPTION

AIMS AND SIGNIFICANCE

Gallbladder (GB) disease is extremely common, affecting 10% to 20% of White adults in the United States with the prevalence rising as high as 70% in specific ethnic groups. [1] Up to 5% of patients with gallstone disease become symptomatic within 1-year. [2] The incidence is greatest among obese people placing patients from racial and ethnic minority groups and those from low-income backgrounds at the greatest risk of disease. [3] Elective cholecystectomy is recommended for patients with symptomatic cholelithiasis or biliary colic. [4] Laparoscopic cholecystectomy is a safe and effective treatment that can be performed in the outpatient setting with complication rates ranging from 0.5-6.0%. [5]

Delayed treatment for symptomatic cholelithiasis and biliary colic often results in acute cholecystitis and related diseases that necessitate emergency surgery. [6] For example, patients waiting 20 weeks or more for elective cholecystectomy were more likely to undergo admission for emergency cholecystectomy than those waiting shorter times. [7] Emergent cholecystectomy results in worse outcomes with almost two-fold higher rates of conversion from laparoscopic to open, increased hospital length of stay by an average of over 5 days, increased rates of morbidity (8.8 vs 3.7%) and mortality (2.6 vs 0.5%), and higher costs (mean charge difference over $6,000) when compared to elective cholecystectomy. [8-10]

Black and Hispanic patients, non-English primary language speakers, and those living in areas of greater social vulnerability are at increased risk of emergent GB surgery when compared with White, English primary language speakers, and those from well-resourced communities. [11-14] Barriers to timely consultation and surgery among historically marginalized communities include patient- and system-level factors. For example, work conflicts, limited paid time off, long clinic wait times, and limited ability to contact the surgical clinic often preclude the ability of patients with social vulnerabilities to seek a consultation or receive elective, operative care.[15-18]

Telemedicine has been shown to save patients in travel time to clinic visits [19], time off work [20], and up to 88 minutes of time during visits [21], when compared to traditional in-person surgical visits. Moreover, patients who have telemedicine visits in surgery have been shown to have similar postoperative complication rates [21, 22], postoperative ED utilization rates, and 30-day readmissions [23] when compared to those with traditional in-person visits. To date, no one has examined the role of telemedicine surgical consultation as a mechanism to promote health equity. Timely consultation with a surgeon to address biliary colic with elective cholecystectomy can reduce the rate of progression of disease and the need for emergent cholecystectomy.

Although early studies suggested disparities in access to telemedicine among marginalized patients, this has been disproven in more recent studies that have shown an increase in the use of telemedicine among Black and socially vulnerable surgical patients [24, 25]. As such, surgical consultations via telemedicine may serve as a promising aid to timely elective cholecystectomy. The Institute of Medicine and National Institutes of Health have established health equity as a critical area for research concentration. In preliminary data, Black patients with biliary colic and symptomatic cholelithiasis treated within the Primary Care Service Line (PCSL) were more likely to present emergently for treatment than White patients. We seek to identify differences in the care paradigms between these populations **to find and test solutions** to surgical disparities within our community. To achieve these goals, we will focus on the following:

Aim 1: Examine racial disparities in outcomes of PCSL patients with symptomatic GB disease with attention to conditional effects of social vulnerabilities. Hypothesis 1.1: Black patients are less likely to undergo cholecystectomy in the elective setting than White patients. Hypothesis 1.2: Time to consultation will modify the effect of race on elective cholecystectomy. Using PennMedicine data we will perform a retrospective cohort study of PCSL patients diagnosed with symptomatic GB disease between Jan. 1 2020 and Sept. 30, 2022. The primary independent variable will be race. Primary outcome will be cholecystectomy. Secondary outcomes will include time from referral to consultation and treatment, completion of consultation, urgency of operative treatment, and cost. Regression models with interaction terms will be used to evaluate effect modification of additional social vulnerability factors (e.g., ethnicity, primary language, insurance, income) on outcomes.

Aim 2: Comparative effectiveness study of telemedicine consultation for symptomatic GB disease in patients with social vulnerability (SV). Hypothesis 2.1: Recruitment will be feasible in the three-month pilot period. Hypothesis 2.2: Time to telemedicine consultation will be shorter than in-person consultation. We will perform a pilot study of telemedicine vs in-person surgical consultation for patients with SV with symptomatic GB disease seen in PCSL clinics between January and June 2023. Rates of surgical consultation will be compared between groups by consultation type. Secondary outcomes will be examined.

Scalability Preliminary data from Penn Chart shows that more than 9,400 PCSL patients seen within the last 6 months have a diagnosis of untreated symptomatic gallbladder disease, and less than 1000 have undergone appropriate treatment (see Figure). This proposal will use a robust approach to explain why patients in our community with symptomatic GB disease do not receive timely treatment (Aim1). Then, we will test the ability of telemedicine to improve timely access to surgical care (Aim2). Currently, PCSL and Emergency Medicine providers refer patients to in-person clinic visits with surgeons. These visits typically require at least a half day off from work, if not more. Since the onset of the pandemic, Penn Medicine surgical clinics have integrated telemedicine into routine practice. Telemedicine decreases time off from work and eliminates the need for transportation (two established barriers to surgical care) while promoting surgical health education, the establishment of trust, and ease of communication with the clinic. Patients will be randomized to a telemedicine preoperative surgical consultation or a traditional in-person surgical consultation. The Penn Medicine system has capacity for this volume of surgical referrals across the three downtown hospitals. Patients will be referred to the location that is most convenient for them. This proposal will pilot the use of telemedicine for patients with symptomatic gallbladder disease treated within the community practices of PennMedicine. We propose that, at scale, this initiative will encourage the **development of a Penn Pathway** for symptomatic cholelithiasis and biliary colic to improve patient and provider satisfaction and will show evidence of PennMedicine's ability to eliminate unnecessary waste in the pathway to surgical health when negotiating **value-based purchasing agreements** with payers.

Equity Plan and Impact
GB disease, associated with obesity, is very common, especially among patients with social vulnerabilities [26, 27]. Guidelines recommend laparoscopic cholecystectomy, a safe and effective same day surgery, for patients with symptomatic cholelithiasis and biliary colic (SGBD). From the literature, we know that delays in care often result in emergent presentation which is significantly associated with increased rates of complications, prolonged hospitalization, and death.[8-10]

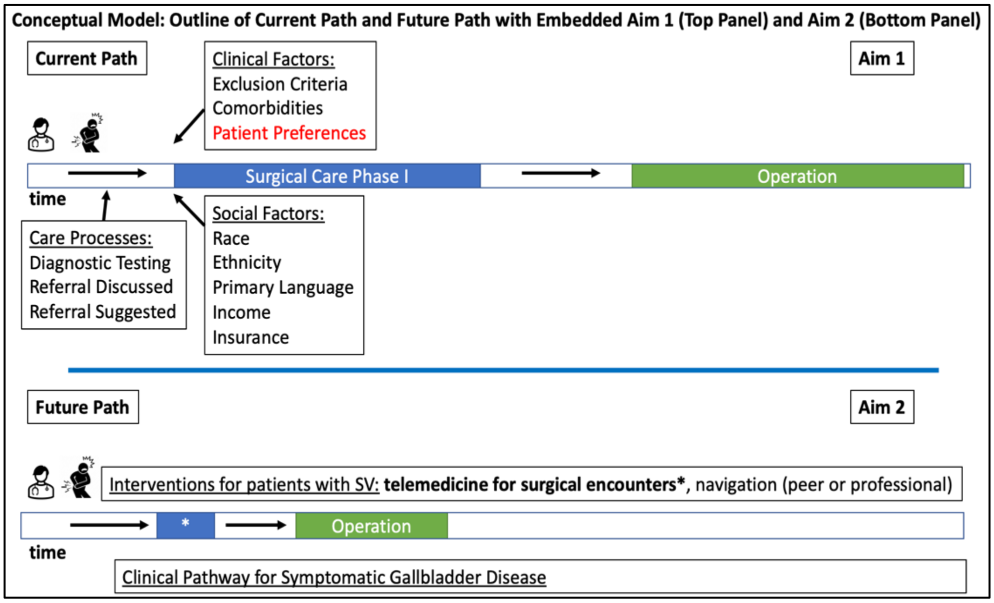

Social vulnerability (SV) is a complex concept with many definitions that are situationally dependent. For this proposal, we define social vulnerability by race, ethnicity, primary language, insurance, and income, five components known to be associated with disparities in care for patients with SGBD. Preliminary data show that the majority of the Penn Medicine PCSL population with SGBD meet our criteria for SV (Black race, 21% (n=1978); Hispanic 6% (n=565); non-English primary language 4% (n=376); low-income neighborhood 91% (n=8573)). Further, in preliminary data, when examining differences across racial groups, for example, Black PCSL patients treated within the past 6 months at PennMedicine were more likely to undergo emergent (vs elective) cholecystectomy than White patients (Black: 65% (76/117); White: 41% (125/303); $p<0.001$). Emergent operations are associated with worse outcomes and greater costs.

Using data abstracted from PennChart, we will conduct an observational study of patients seen within the PCSL and the those discharged from the Emergency Department with symptomatic cholelithiasis and biliary colic between Jan. 1, 2020 and Sept. 30, 2022. These data will allow us to examine quality of care in two dimensions, safety and efficiency, for patients treated in the community setting. While we focus our analysis on race, we will study the conditional effects of other dimensions of SV on quality. **To date, the reasons for and impact of delays in treatment of symptomatic GB disease in Black patients and those with other SV within our PennMedicine community is unknown.** To test a solution to the problems of time off work and transportation, we will enroll patients with SV seen in the PCSL clinics in a pilot designed to test telemedicine as a mechanism to improve timely access to surgical care. Health equity will be measured by rates of surgical consultation among patients with SV and timeliness of surgical consultation and treatment.

RESEARCH PLAN AND METHODOLOGY

Overall Approach To address surgical health equity in patients with gallbladder disease, we will: 1) use regression models with interaction terms to examine racial disparities in surgical consultation and the conditional effects of SV factors on this relationship, 2) examine the marginal effects of social factors on outcomes, and 3) test a potential solution to surgical disparities, telemedicine. The first aim will be conducted in a retrospective observational study. Aim two will be performed as a pilot study of Penn Primary Care Service Line patients with SV. For this aim, we will pragmatically assign two groups to traditional care and telemedicine consultation and study the resultant outcomes. (See Conceptual Model above)

Research Team Our team has expertise in health services research, disparities research, general surgery and primary care. To conduct this innovative, scientifically rigorous mixed-methods study, we will also employ the services of research coordinators (DOS Clinical Trials Unit), data scientists (LDI-HEDAP), and statisticians (Center for Surgery and Health Economics). To ensure that the study will be conducted in a way that is acceptable to the community, we will recruit two patients from the PCSL to serve as advisors. Advisors will be compensated at $50 per hour for an estimated total of 8 hours of work.

Impact This proposal has the potential to scale across practices, influence value-based purchasing agreements and enhance patient and clinician satisfaction. Moreover, the proposed aims will have a major impact on health equity by: 1) assessing the clinical and financial costs of delays, 2) identifying barriers to timely treatment, 3) testing a simple intervention to improve access to surgical health equity for patients with social vulnerabilities.

Outcomes of PCSL Patients with Symptomatic Gallbladder Disease (AIM 1)

Design A retrospective cohort study.

Figure. Primary Care Service Line (PCSL) Catchment Area

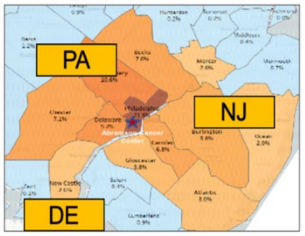

Study Subjects and Eligibility Criteria Patients with a diagnosis of symptomatic cholelithiasis or biliary colic who were >18 years of age and enrolled in the Primary Care Service Line (PCSL) Registry in Clarity or evaluated and discharged from the Emergency Department (PAH, PPMC and HUP) between Jan. 1 2020 and Sept. 30, 2022 will be eligible for inclusion (data will be obtained for January 1 2020 to Dec. 31, 2022). The Primary Care Service Line (PCSL) covers more than 500,000 patients across the Penn Medicine catchment area including 12 contiguous urban, suburban, and rural counties in the tri-state area of PA, NJ, and DE (See Figure). The population is approximately 37.6% Black and 7.5% underinsured. Symptomatic cholelithiasis and biliary colic will be identified using International Classification of Diseases, Tenth Revision, Clinical Modification codes (ICD-10-CM). A 6-month "look-back" will be performed to confirm the first instance of disease. Patients with the following criteria will be excluded from study: 1) history of prior cholecystectomy 2) prisoners, or 3) contraindication to cholecystectomy including GB cancer, advanced cirrhosis and untreated coagulopathy.

Data Sources and Elements Clinical data will be abstracted from Clarity and financial data from the Horizon Performance Manager (HPM) dataset. This will allow real-world calculations of the value of care at Penn Medicine.

Definition of Social Vulnerability: Race will be the primary independent variable, given the established association between race and surgical disparities in the literature and preliminary data from Penn Chart. Ethnicity (Hispanic/non-Hispanic), primary language (English/non-English), insurance (Medicaid, Medicare, private, other) and low-income neighborhood (home zip code) will be identified in Clarity.
Covariates: Age, sex, comorbidities (defined by Elixhauser et al [28] using the index PCP visit and a 6-month look-back period), history of cholecystectomy, GB cancer, cirrhosis, coagulopathy and diagnostic imaging (US and CT SCAN) information will be obtained from Clarity. ICD-10 diagnosis codes will be used for clinical diagnoses. Date diagnosis add to chart, date of confirmatory imaging, date of discussion of treatment, date of referral to a surgeon and prisoner status will be obtained via direct chart review of PCSL notes within the 3-months following the initial diagnosis of SGBD. Access point will be designated as PCSL or Emergency Dept..
Clinical Outcomes: Surgical consultation will be obtained from Horizon Performance Manager (HPM) via CPT codes for consultation. [29] Operative treatment will be determined using CPT and ICD-10-PCS codes for cholecystectomy as listed in HPM (CPT 47562, CPT 47563, CPT 47600, CPT 47605, CPT 56340, CPT 56341, ICD10 0FB40ZX, ICD10 OFB40ZZ, ICD10 OFT40ZZ) and operative urgency (emergent/urgent vs elective) will be based on the admission source for the associated operative encounter. We hypothesize that Black patients are less likely to undergo cholecystectomy in the elective setting than White patients (H1.1), that time to

consultation will modify the effect of race on elective cholecystectomy (H1.2), and that underinsurance (e.g., Medicaid) will exacerbate the effect of race on outcomes.

Financial Outcomes: We will assign costs using data from HPM for billable encounters in the emergency department, inpatient setting and ambulatory procedure units with a primary diagnosis related to symptomatic cholelithiasis and biliary colic within 90-days of diagnosis. Permutations of cost will be assessed with nonparametric tests and regression. We hypothesize that costs will be higher for Black patients and impacted by other SV factors (H1:4). Stratified analysis will be performed by treatment group and point of access.

Analytic Strategy In order to adjust for potential confounders, multivariable regression (logistic and robust) will be used to explain the relationship between race and outcomes. Interaction terms will be used to examine effect modification of social vulnerability factors on the relationship between race and outcomes. The marginal effects of social vulnerability factors will be calculated. All analysis will be performed using STATA Version 17 (Stata Corporation, College Station, TX).

PRELIMINARY STUDY RESULTS and POWER CALCULATIONS

Our preliminary data from Penn Chart demonstrate racial disparities in access to care with a greater proportion of Black PCSL patients undergoing emergent cholecystectomy than White patients (see Equity section). We will be powered at 80% with an alpha of 0.05 to detect differences between Black and White patient outcomes as small as 5%. Note that preliminary data shows a difference of 24% in emergent chole.

Telemedicine Consultation as a Mechanism to Improve Surgical Health Equity in Benign Disease (AIM 2)

Design Pragmatic pilot trial.

Setting and Participants Patients ages 18+ years with symptomatic cholelithiasis or biliary colic who are a member of a racial or ethnic minority, not primary English speaking, from a low-income neighborhood or underinsured will be identified from the PCSL (see above) and Emergency Department (ED) discharges including referral to surgery. See **Figure** for exclusion criteria. After Institutional Review Board (IRB) approval, potential participants with a new ICD-10 code for symptomatic cholelithiasis or biliary colic seen by a PCSL provider between April 1, and June 30, 2023 will be identified. The diagnosis and discussion with the PCSL provider will be confirmed by chart review to ensure the patient is aware of their diagnosis. Then, patients will be recruited via the electronic health record. Nonresponses will be contacted by phone. If enrollment is lower than required, patients will be approached about participation at the time of the in-person PCSL or ED visit. Participation will be voluntary and informed consent will be obtained. For this pilot study, enrollment will proceed to a maximum of 25 per group. We anticipate between 2-37 eligible patients per week. Participants will receive an incentive of $20 for participation. Demographics and clinical information will be collected in REDCap, a HIPPA-compliant, secure web application for building and managing online surveys and databases.

Exposure, Intervention Assignment, Covariates and Outcomes

Exposure: Assignment to telemedicine surgical consultation (vs. usual care) during the week of diagnosis of symptomatic cholelithiasis or biliary colic.

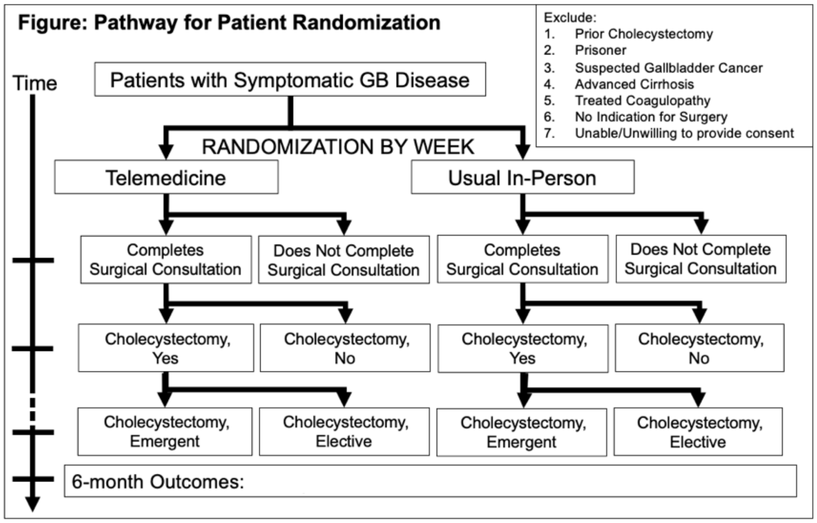

Intervention Assignment: Pragmatic randomization will be performed by group enrollment in alternating weeks.

Covariates: Participants will be followed for 6-months from enrollment. Data on patient attributes will include age, race, sex, insurance status, primary language and 9-digit zip code as a proxy for neighborhood income level. Clinical factors will be abstracted from the electronic medical record including comorbid conditions as defined in the Elixhauser Indices [28], bilirubin, liver function studies, ultrasound scan and CT scan results.

Outcomes: Primary outcome will be proportion of patients who undergo surgical consultation. Surgical consultation will be determined via chart review including care everywhere to

ensure capture of visits outside of Penn Medicine. Secondary outcomes will include time to consultation, proportion to undergo cholecystectomy, time to surgery, and emergent/urgent vs elective operation.

Data Analysis Descriptive statistics will be performed. Univariate comparisons to test hypotheses will be performed using the chi-square test or Fisher's exact test for categorical data and t-test or Wilcoxon rank-sum for continuous data. Statistical analysis will be performed using STATA Version 17 (Stata Corporation, College Station, TX). Stratified analyses will be performed by race, ethnicity, primary language, median neighborhood income, and insurance status as permitted by sample size.

PRELIMINARY STUDY RESULTS and POWER CALCULATIONS

In addition to providing preliminary evidence of the efficacy potential of telemedicine surgical consultations, the goal of the pilot study is to estimate recruitment and retention rates and population variance of social vulnerability factors. Because we want to be powered to detect small effect sizes, ($0.1 \le d < 0.3$) where d is the standardized difference, we will target enrollment of 20 participants per group to achieve a power of 80%, $\alpha = 0.05$. [32]

Limitations

- The study focuses on patients already connected to primary care providers. Many patients with SV may not reach that point in the health system. However, in clinical practice at Penn Medicine it is not uncommon for patients to have insurance that allows primary care at our community sites without providing benefits for surgical care for nonemergency conditions. This approach to patient care exposes patients treated in our community to the excessive clinical and financial risks associated with emergency surgery. Because we will conduct an explanatory analysis of factors associated with surgical consultation and treatment, we will identify barriers to surgical health equity (e.g., insurance coverage) within our system. This will allow us to develop strategies to achieve health equity at the system level.
- In Aim 1, we will not be able to assess patient preference as a reason for undertreatment. We will be able to identify major structural barriers to care (e.g., primary language, insurance etc.). In the pilot study, we will include items on patient preferences (e.g., refusal to see a surgeon or have an operation). This will allow us to gauge the magnitude of this problem in patients with symptomatic GB disease at Penn Medicine. If this is a big problem, we will have to perform additional qualitative studies to determine optimal solutions.
- Telehealth consultation may not be available to the most vulnerable patients. While that was true historically, during the pandemic studies confirmed that telehealth was used widely, especially among those with social vulnerabilities. Moreover, prior to the pandemic, surgical telehealth was used effectively in our system. In the original study, 79% of patients were able to receive telemedicine surgical visits and only 3% were unable due to language or communication barriers.

Future Directions

This study will provide data to develop targeted solutions to achieve surgical health equity at Penn Medicine. For example, in Aim 1, if we discover that underinsurance is the large barrier to treatment, then expanded professional navigation may be the way forward. If mistrust of the system and fears of surgery emerge as barriers to treatment, then peer navigation may be important. Alternatively, if we find that time off work and transportation to visits preclude surgical treatment, then telemedicine consultations (and resultant elective same day surgery rather than emergency in-patient care), may lead us to the path to health equity. We will test this directly in Aim 2. Improving access to care will result in improved patient and provider satisfaction. We will use this information to convene stakeholders within the system to build a Penn Pathway for symptomatic cholelithiasis and biliary colic. Further, this information will provide direction for the design of a larger trial to test multiple additional approaches to overcome barriers to surgical health equity within Penn Medicine that can be scaled across practices and even beyond the confines of our own network. These data will set the stage for high value care bundles to treat patients with social vulnerabilities within Penn Medicine.

REFERENCES

1. Cox, M.R., G.D. Eslick, and R. Padbury, *The management of gallstone disease: a practical and evidence-based approach*. 2018: Springer.
2. Altieri, M.S., et al., *What happens to biliary colic patients in New York State? 10-year follow-up from emergency department visits.* Surgical Endoscopy, 2018. **32**(4): p. 2058-2066.
3. Prevention, C.f.D.C.a. *Adult Obesity Facts*. Division of Nutrition, Physical Activity, and Obesity, National Center for Chronic Disease Prevention and Health Promotion 2022 May 17, 2022 [cited 2022 September 19, 2022]; Available from: https://www.cdc.gov/obesity/data/adult.html#:~:text=Obesity%20affects%20some%20groups%20more%20than%20others&text=Non%2DHispanic%20Black%20adults%20(49.9,Hispanic%20Asian%20adults%20(16.1%25).%20Accessed%2009/18/2022.
4. Overby, D., et al., *Society of American Gastrointestinal and Endoscopic Surgeons (SAGES). SAGES guidelines for the clinical application of laparoscopic biliary tract surgery.* Surg Endosc, 2010. **24**(10): p. 2368-2386.
5. Radunovic, M., et al., *Complications of laparoscopic cholecystectomy: our experience from a retrospective analysis.* Open access Macedonian journal of medical sciences, 2016. **4**(4): p. 641.
6. Cheruvu, C. and I. Eyre-Brook, *Consequences of prolonged wait before gallbladder surgery.* Annals of the Royal College of Surgeons of England, 2002. **84**(1): p. 20.
7. Sobolev, B., et al., *Risk of emergency admission while awaiting elective cholecystectomy.* Cmaj, 2003. **169**(7): p. 662-665.
8. To, K.B., et al., *Emergent versus elective cholecystectomy: conversion rates and outcomes.* Surgical infections, 2013. **14**(6): p. 512-519.
9. Rutledge, D., D. Jones, and R. Rege, *Consequences of delay in surgical treatment of biliary disease.* The American journal of surgery, 2000. **180**(6): p. 466-469.
10. Muñoz, E., et al., *Surgonomics: The cost of cholecystectomy.* Surgery, 1984. **96**(4): p. 642-647.
11. Carmichael, H., et al., *Using the social vulnerability index to examine local disparities in emergent and elective cholecystectomy.* Journal of Surgical Research, 2019. **243**: p. 160-164.
12. Lin, J.A., et al., *Pandemic Recovery: Persistent Disparities in Access to Elective Surgical Procedures.* Annals of Surgery, 2021.
13. Shenoy, R., et al., *Symptomatic Cholelithiasis: Do Minority Patients Experience Delays to Surgery?* Journal of Surgical Research, 2022. **272**: p. 88-95.
14. Janeway, M.G., et al., *Disparities in Utilization of Ambulatory Cholecystectomy: Results From Three States.* Journal of Surgical Research, 2021. **266**: p. 373-382.
15. Saluja, S., et al., *Community-Based Health Care Navigation's Impact on Access to Primary Care for Low-Income Latinos.* The Journal of the American Board of Family Medicine, 2022. **35**(1): p. 44-54.
16. Han, H.-R., et al., *Tailored lay health worker intervention improves breast cancer screening outcomes in non-adherent Korean-American women.* Health education research, 2009. **24**(2): p. 318-329.
17. Chavarri-Guerra, Y., et al., *Patient navigation to enhance access to care for underserved patients with a suspicion or diagnosis of cancer.* The oncologist, 2019. **24**(9): p. 1195-1200.
18. Berkowitz, R.L., et al., *Patient Experiences in a Linguistically Diverse Safety Net Primary Care Setting: Qualitative Study.* Journal of Participatory Medicine, 2018. **10**(1): p. e9229.
19. Gunter, R.L., et al., *Current use of telemedicine for post-discharge surgical care: a systematic review.* Journal of the American College of Surgeons, 2016. **222**(5): p. 915.
20. Sathiyakumar, V., et al., *Prospective randomized controlled trial using telemedicine for follow-ups in an orthopedic trauma population: a pilot study.* Journal of orthopaedic trauma, 2015. **29**(3): p. e139-e145.
21. Williams, A.M., et al., *The role of telemedicine in postoperative care.* Mhealth, 2018. **4**.
22. Hwa, K. and S.M. Wren, *Telehealth follow-up in lieu of postoperative clinic visit for ambulatory surgery: results of a pilot program.* JAMA surgery, 2013. **148**(9): p. 823-827.
23. Abbitt, D., et al., *Telehealth follow-up after cholecystectomy is safe in veterans.* Surgical Endoscopy, 2022: p. 1-7.
24. Dekker, P.K., et al., *Telemedicine in the wake of the COVID-19 pandemic: increasing access to surgical care.* Plastic and Reconstructive Surgery Global Open, 2021. **9**(1).
25. Eruchalu, C.N., et al., *Demographic disparity in use of telemedicine for ambulatory general surgical consultation during the COVID-19 pandemic: Analysis of the initial public health emergency and second phase periods.* Journal of the American College of Surgeons, 2022. **234**(2): p. 191-202.

26. Maurer, K.R., et al., *Prevalence of gallstone disease in Hispanic populations in the United States.* Gastroenterology, 1989. **96**(2): p. 487-492.
27. Shaffer, E.A., *Epidemiology of gallbladder stone disease.* Best practice & research Clinical gastroenterology, 2006. **20**(6): p. 981-996.
28. Elixhauser, A., et al., *Comorbidity measures for use with administrative data.* Medical care, 1998: p. 8-27.
29. Kaufman, E.J., et al., *Operative and Nonoperative Outcomes of Emergency General Surgery Conditions: An Observational Study Using a Novel Instrumental Variable.* Annals of Surgery, 2022.
30. Eypasch, E., et al., *Gastrointestinal Quality of Life Index: development, validation and application of a new instrument.* Journal of British Surgery, 1995. **82**(2): p. 216-222.
31. Schmidt, M., et al., *Post-cholecystectomy symptoms were caused by persistence of a functional gastrointestinal disorder.* World journal of gastroenterology: WJG, 2012. **18**(12): p. 1365.
32. Bell, M.L., A.L. Whitehead, and S.A. Julious, *Guidance for using pilot studies to inform the design of intervention trials with continuous outcomes.* Clinical epidemiology, 2018. **10**: p. 153.
