# Supplementary material for: Telemedicine and Access to Elective Cholecystectomy for Socially Vulnerable Adults: A Pilot Randomized Clinical Trial
Source: JAMA Netw Open. 2024 Oct 9;7(10):e2438137. doi: 10.1001/jamanetworkopen.2024.38137 (PMC11581524; doi:10.1001/jamanetworkopen.2024.38137)
Supplement: Supplement 2. — Data Sharing Statement [file jamanetwopen-e2438137-s002.pdf]

Data Sharing Statement

Bakillah. Telemedicine and Access to Elective Cholecystectomy for Socially Vulnerable Adults. *JAMA Netw Open*. Published October 09, 2024. doi:10.1001/jamanetworkopen.2024.38137

Data
Data available: No
